# Multifunctional Chiral Three-Dimensional Phosphite Frameworks Showing Dielectric Anomaly and High Proton Conductivity

**DOI:** 10.3389/fchem.2021.778687

**Published:** 2021-12-09

**Authors:** S. S. Yu, C. Y. Xu, X. Pan, X. Q. Pan, H. B. Duan, H. Zhang

**Affiliations:** ^1^ School of Environmental Science, Nanjing Xiaozhuang University, Nanjing, China; ^2^ Key Laboratory of Advanced Functional Materials of Nanjing, Nanjing Xiaozhuang University, Nanjing, China

**Keywords:** phosphite frameworks, dielectric response, pron conductor, molecular motions, ionothermal method

## Abstract

Chair 3D Co(II) phosphite frameworks have been prepared by the ionothermal method. It belongs to chiral space group P3_2_21, and the whole framework can be topologically represented as a chiral 4-connected qtz net. It shows a multistep dielectric response arising from the reorientation of Me_2_-DABCO in the chiral cavities. It can also serve as a pron conductor with high conductivity, 1.71 × 10^−3^ S cm^−1^, at room temperature, which is attributed to the formation of denser hydrogen-bonding networks providing efficient proton-transfer pathways.

## Introduction

Crystalline materials with open frameworks, such as zeolites and metal-organic frameworks (MOFs), containing a rotatable guest component, have attracted considerable attention owing to their sensitive and controllable responses to external stimuli, such as thermal, pressure, light, or electricity (Kay et al., 2007; Jin et al., 2011; Yan et al., 2012; Hu and Liu, 2014; Huang and Isaacs, 2014; Du et al., 2015). Guest component motion and crystalline environments have paved the way for controlling the molecular dynamics in crystals by designing crystal structures (Harada et al., 2015; Zhao et al., 2021). Metal phosphate-based open frameworks have excellent chemical and thermal stabilities compared to MOFs (Xu et al., 1996; Yuan et al., 2000; Chippindale et al., 2003; Matsuda et al., 2013; Hao et al., 2022). A distinct feature of metal phosphate frameworks is the rigidity; in contrast, the flexibility frameworks of MOFs are strongly related with the nature of the organic linker (Kitagawa et al., 2004; Long and Yaghi, 2009). Probing the local dynamics enables vast opportunities to reveal the response of the rigid frameworks to the presence of guest molecules. For instance, guest molecular Emim^+^ ion motions in channels formed by rigid frameworks can give rise to temperature-dependent conductivity properties (Emim^+^ = 1-ethyl-3-methylimidazolium) (Chen et al., 2011). [(CH_3_)_2_NH_2_][MII(HCOO)_3_] (M = Mn, Fe, Co, Ni, Zn) exhibit a paraelectric–ferroelectric phase transition due to the disorder–order transition of the guest [(CH_3_)_2_NH_2_]^+^ cation (Jain et al., 2009; Fu et al., 2011). [Him]_2_[KFe(CN)_6_] (Him = imidazolium cation) shows striking dielectric switch properties owing to the disorder–order transition of the guest molecules (Zhang et al., 2010). However, only limited types of molecular motions have been explored, aiming for the development of functional materials with the desired properties (Wang et al., 2021; Zhao and Xu, 2021).

Meanwhile, the three-dimensional (3D) crystal lattice can provide well-designed pores for the proton conduction pathway, and the various interactions between the pores and guest molecules (Zhu et al., 2014; Meng et al., 2015), such as hydrogen bonding and weak Coulombic interaction, may contribute to the introduction of guest as media into the pores for proton conduction (Horike et al., 2012; Liu et al., 2015; Ye et al., 2015). At present, MOF-based proton conductors have been extensively investigated due to significant advantages; for example, the pore size is designable and controllable, and the large pores favor the increase in the movement of proton carriers which improves proton conductance characteristics (Tang et al., 2014). However, the poor stability to water is an obvious drawback for MOF proton conductors, and this is due to the weak coordination bonds between the nodes and organic linker. Metal phosphate frameworks are one of the best candidates in new types of proton conduction materials because the porous frameworks are built from robust inorganic units.

In this work, we try to introduce rotatable 1,4-diazabicyclo(2.2.2)octane (DABCO) as guest component into the inorganic lattice. A chiral 3D metal phosphite framework with a formula [(Me_2_-DABCO)][Co_2_(HPO_3_)_3_] (compound **1**) [Me_2_-DABCO = N,N′-dimethyl-1,4-diazabicyclo(2.2.2)octane] was obtained by the ionothermal method. It is notable that the guest (Me_2_-DABCO)^2+^ cations were generated *in situ via* an alkylation reaction of DABCO and dimethyl phosphites. Compound **1** shows the multistep dielectric response and high proton conductivity, 1.71 × 10^−3^ S cm^−1^, under 99% RH even at room temperature.

The compound **1** crystal was synthesized by reaction CoCl_2_ 6H_2_O with dimethyl phosphite and DABCO in melting choline chloride/1,3-dimethylurea (see Supplementary Material). The synthesis method is similar with the reported work by Zhang and coworkers (Li et al., 2013). The phase purification of the as-prepared sample was characterized by power X-ray diffraction (PXRD) and IR spectroscopy (Supplementary Figures S1, S2). **1** exhibits high thermal stability. TG analysis its stability up to 330°C (Supplementary Figure S3).

## Results and Discussion

### Crystal Structure

**FIGURE 1:**
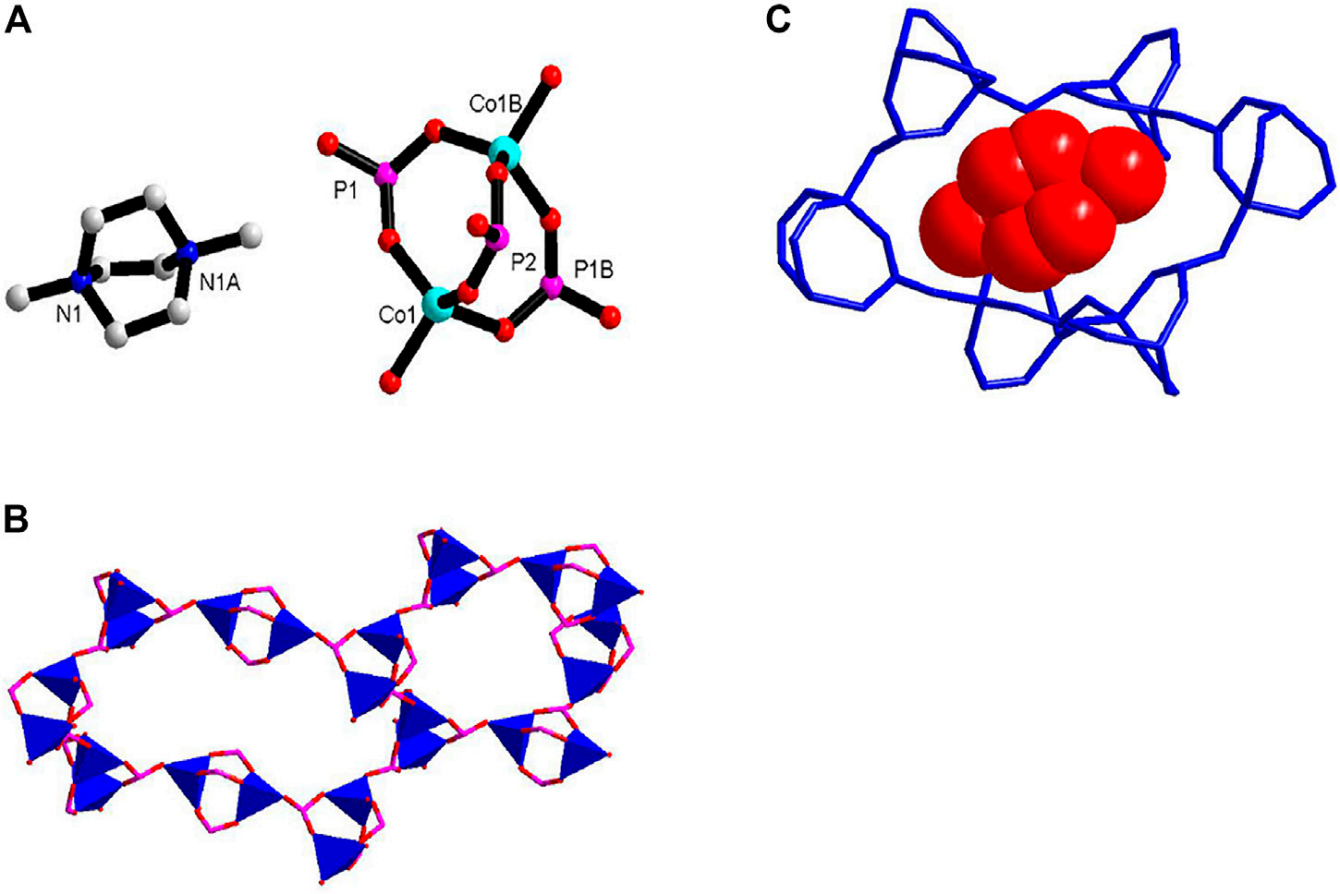
**(A)** The coordination geometry of **1** showing [Co_2_(HPO_3_)_3_] SBU; **(B)** 3D framework structure showing two types of chiral nanometer-sized cavities; and **(C)** close-up of the Me_2_-DABCO rotator in the crystal, shown in the red space filling model; rigid stator illustrated in blue stick.

Crystal of **1** at 100 K belongs to chiral space group *P3*_*2*_ *21.* The lattice parameters are very similar with the reported compound (Li et al., 2013). There is one crystallographically independent Co^2+^ and two different P^3+^ centers in an asymmetric unit (Figure 1A). The Co^2+^ ions are coordinated by four oxygen atoms from four HPO_3_^2−^ ligands in tetrahedral geometry. Each P2 atom is connected by two CoO_4_ tetrahedrons, and the P1 atom is connected by three CoO_4_ tetrahedrons. The existence of the P–H bond is proved by the IR spectrum (Supplementary Figure S2) and other reported compounds (Wang et al., 2013; Wang et al., 2014). [Co_2_(HPO_3_)_3_] SBU (SBU = secondary building units) were formed and linked with four adjacent SBU forming 3D open frameworks. The whole framework of **1** can be topologically represented as a chiral 4-connected qtz net. Two types of chiral nanometer-sized negative cavities were formed (Figure 1B). The (Me_2_-DABCO)^2+^ cations reside in the cavities as charge compensation. **1** can serve as an amphidynamic molecular machine (Figure 1C). The inorganic phosphite frameworks are the rigid stator, Me_2_-DABCO is the rotator, and the weak charge-assisted hydrogen bonding is the rotation axis. The rotational motion of Me_2_-DABCO in cavities can be reflected in dielectric investigation.

### Dielectric Properties

**FIGURE 2:**
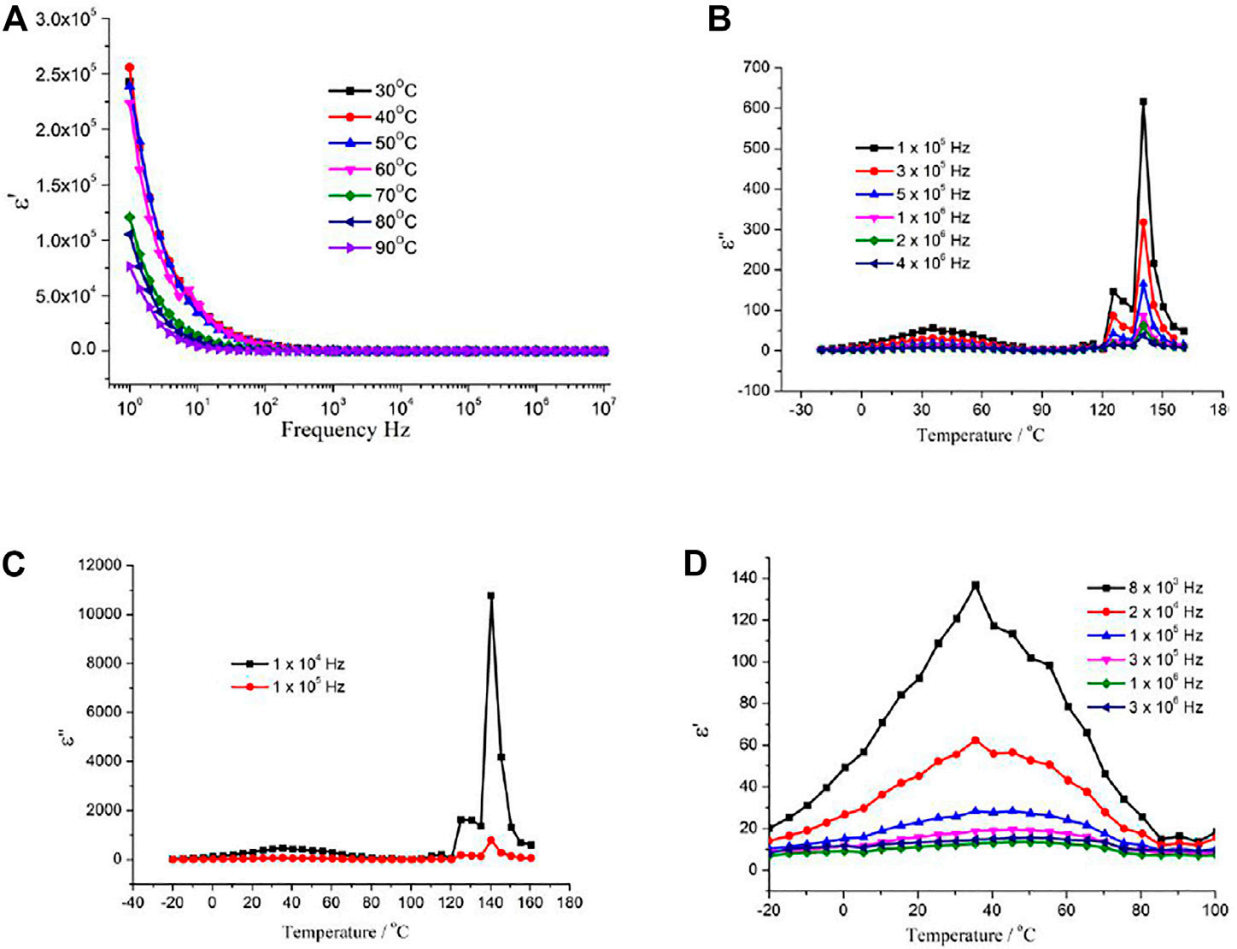
**(A)** Frequency dependencies of ε′ in the 30°C–90°C temperature range; **(B)** and **(C)** temperature dependencies of ε′ in the selected frequency range; and **(D)** temperature dependencies of ε′ shows a broad dielectric peak below 100°C of **1**.

Dielectric responses due to molecular reorientation of Me_2_-DABCO were observed for the crystal of **1**. The frequency dependence of the dielectric permittivity real part (ε′) and imaginary part (ε′′) is shown in Figure 2A and Supplementary Figure S4 in the temperature range 30°C–90°C and at the frequency range of 1–10^7^ Hz. A strong low-frequency dispersion of ε′ and ε″ is shown. The ε′ and ε″ values rapidly decrease with frequency increase. In general, the rate of molecular reorientation is higher than the applied electric field at low frequency; the dipole motion can follow the switching of the electric field, and the dielectric constant value is large. On the contrary, in the high frequency, the dipole motion is too slow to follow the electric field and is regarded as stationary. The dielectric constant value is small. When the rate of molecular motion is compared to the frequency of the applied electric field, the molecular dipole orientational polarization can produce a phase lag and result in the absorption of energy by the materials. Figure 2B shows the temperature-dependent dielectric constant imaginary part at the selected frequency. Two obvious dielectric peaks were observed at 125°C and 140°C at f = 10^4^ Hz (Figure 2C), respectively. Two dielectric peaks were independent on the frequency of the electric field (Figure 2B). The observed dielectric peaks can be attributed to the reorientation of the Me_2_-DABCO. Below 100°C, the reorientation of Me_2_-DABCO was slower than the applied frequency and became fast when the temperature is higher than 120°C. Compared with our previous work, the reorientation-activated temperature of Me_2_-DABCO is higher than that of DABCO in the phosphite framework (Wang et al., 2013; Wang et al., 2014), which can be ascribed to the large molecular structure in **1**. Furthermore, **1** exhibits an unusually high dielectric constant compared to the perovskite-based ferroelectric BaTiO_3_, Pb (Zr,Ti)O_3_, and (Ba,Sr)TiO_3_ (ε′ > 10^3^) and the body-centered cubic compound CaCu_3_Ti_4_O_12_ (ε′ > 10^4^). It is noted that a broad dielectric peak was appeared center at ca. 45°C at f = 10^5^ Hz (Figure 2D). The peak position of the maximum is slightly changed with the frequency change and the signal increase with decreasing frequency between 10^3^ and 10^6^ Hz (Figure 2D), which is a typical dielectric relaxation and may be ascribed to local charge order.

### Proton Conductance

**FIGURE 3:**
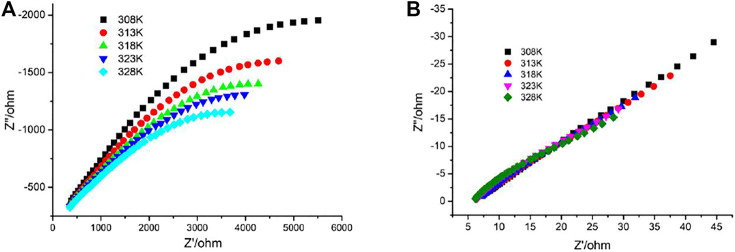
Nyquist plots of **1** at **(A)** 75% and **(B)** 99% RH at the selected temperatures.

The presence of 1D channels include the -POH groups pointing toward the interior of the channels in the crystal structure of **1** which makes them good candidates as proton conductors. Alternating current (AC) impedance measurements under different humid conditions were performed. The proton conductivities of **1** were calculated from the fitting of the Nyquist plots. Compared with the proton conductivity of the single crystal sample, the impedance plots for the pellet sample could be fitted by two serial equivalent RC circuits, in which one RC circuit was assigned to the bulk phase, the other to the grain boundaries (Barsoukov and Macdonald, 2005; Ogiwara et al., 2016). The relative humidity (RH) dependence of proton conductance was investigated in the temperature range of 25°C–45°C for **1**. The AC impedance measurements were performed at 60%, 75%, and 99% RH, and the corresponding Nyquist plots are shown in Supplementary Figure S5, Figures 3A,B. The conductivities for **1** at the selected temperature and RH are shown in Table 1. Under 60% RH, the proton conductivities for **1** at 25°C and 45°C were 1.46 × 10^−5^ and 2.36 × 10^−5^ S cm^−1^, respectively. Under 99% RH, the proton conductivities for **1** at 25°C and 45°C were 1.71 × 10^−3^ and 5.56 × 10^−3^ S cm^−1^, respectively. Such high conductivities are larger than 1-2 orders of magnitude; these values were reported for some metal phosphonate frameworks with *σ* = (3.5–5) 10^−5^ S cm^−1^ at 25°C and 95%–98% RH (Taylor et al., 2010; Costantino et al., 2012). In particular, the value of 5.56 × 10^−3^ S cm^−1^ is slightly higher than that of conductivity reported among MOF materials, such as those of Cu-DOSA (1.9 × 10^−3^ S cm^−1^ at 85°C and 98% RH) (Dong et al., 2013), PCMOF-5 (2.5 × 10^−3^ S cm^−1^ at 60°C and 98% RH) (Meng et al., 2015), and In-IA-2D-1 (3.4 × 10^−3^ S cm^−1^ at 27°C and 98% RH) (Panda et al., 2013; Taylor et al., 2013). The variation of proton conductivity with the increase in RH indicates that the process of proton conduction in **1** is dominated by water-mediated proton conduction, which may be because the formation of the H-bond network between water molecules and the framework provides efficient proton transport pathways.

**TABLE 1:** Proton conductivity data of **1** (S cm^−1^).

Temperature/°C	Conductivity		
	RH = 60%	RH = 75%	RH = 99%
25	1.46 × 10^−5^	7.39 × 10^−5^	1.71 × 10^−3^
30	1.73 × 10^−5^	9.34 × 10^−5^	2.22 × 10^−3^
35	1.94 × 10^−5^	1.11 × 10^−4^	3.25 × 10^−3^
40	2.08 × 10^−5^	1.32 × 10^−4^	4.66 × 10^−3^
45	2.36 × 10^−5^	1.43 × 10^−5^	5.56 × 10^−3^

The proton transport activation energy, *E*_*a*_, was estimated using the Arrhenius equation,

$$\ln\left(\sigma T\right)=\ln A-\frac{E_{a}}{K_{B}T}$$

**FIGURE 4:**
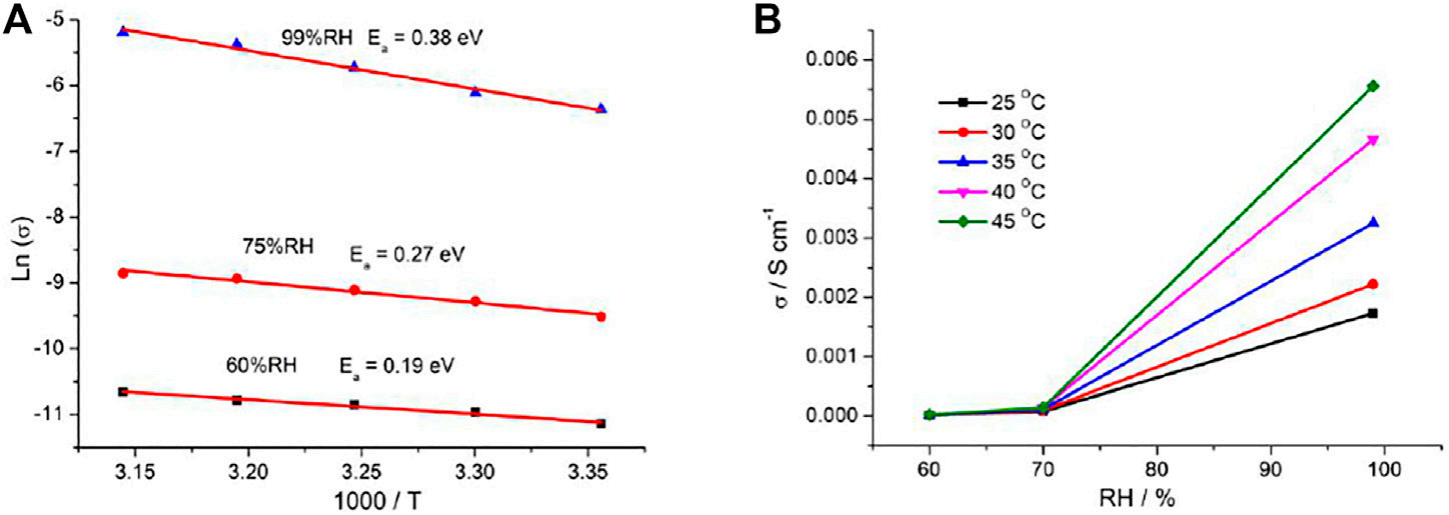
**(A)** Plots of ln(*σ*) vs. 1000/*T* at selected RH and **(B)** plots of *σ* vs. RH at the selected temperatures for **1**.

Plots of ln(*σ*) versus 1000/*T* at selected RH are displayed in Figure 4A, and the RH dependences of conductivity at the selected temperature are shown in Figure 4B. The activation energy (*E*_*a*_) can be obtained according to the linear fitting slope. Under 60%, 75, and 99% RH, *E*_*a*_ values are 0.19, 0.27, and 0.38 eV, respectively. In the channels of porous coordination polymers, generally, the proton transport is achievable *via* two types of mechanisms termed Grotthuss mechanism (*E*_*a*_ < 0.4 eV) and vehicle mechanism (*E*_*a*_ > 0.4 eV). For the Grotthuss mechanism, the proton transports between the relatively stationary host anions. The vehicle mechanism usually restricted to materials with open structures to allow passage of large ions and molecules. For **1**, the large-volume Me_2_-DABCO ions in channels block the migration of the proton; additionally, the long-range hydrogen bonding interaction was perfect between water molecules and stationary inorganic frameworks. Combined with the above analysis and our experimental results, the proton transport is mainly attributed to the Grotthuss mechanism in **1**.

## Conclusion

In summary, we successfully prepared chair 3D Co(II) phosphite frameworks and explored their dielectric properties and proton conduction behavior underwater. Two obvious dielectric peaks were observed at 125°C and 140°C, respectively, which can be attributed to the reorientation of Me_2_-DABCO in the chiral cavities. The proton conductivity was investigated at different RH, the results indicating that the conductivity is enhanced by 2 orders of magnitude at the same temperature with the RH increased to 99%. This compound shows high proton conductivity, 1.71 × 10^−3^ S cm^−1^, under 99% RH even at room temperature, which is attributed to the formation of denser hydrogen-bonding networks providing efficient proton-transfer pathways. This study reveals that some intriguing properties could be obtained by introducing rotatable components into channels, and provides a novel insight into the design and synthesis of multifunctional metal phosphate frameworks.

## Notes

Crystal data for compound **1** (CCDC number 2100996), space group *P3221*, *a* = 9.8280(3)Å, *b* = 9.8280(3)Å, *c* = 15.8057(7)Å, *α* = *β* = 90.00°, *γ* = 120.00°, V = 1322.13(10)Å^3^, Z = 3, D_c_ = 1.873 g cm^−3^.

## Data Availability

The datasets presented in this study can be found in online repositories. The names of the repository/repositories and accession number(s) can be found in the article/Supplementary Material.
