# Supplementary material for: Multifunctional Chiral Three-Dimensional Phosphite Frameworks Showing Dielectric Anomaly and High Proton Conductivity
Source: Front Chem. 2021 Dec 9;9:778687. doi: 10.3389/fchem.2021.778687 (PMC8695548; doi:10.3389/fchem.2021.778687)
Supplement: Supplementary file 1 [file Table1.DOCX]

Multifunctional chiral three-dimensional phosphite frameworks showing dielectric anomaly and high proton conductivity

S. S. Yu,^a^ C. Y. Xu,^a^ X, Pan,^a^ X. Q. Pan,^a^ H. B. Duan*^a^ and H. Zhang*^a^

^a^ School of Environmental Science, Nanjing Xiaozhuang University, Nanjing 211171, P. R. China

^b^ Key laboratory of Advanced Functional Materials of Nanjing, Nanjing Xiaozhuang University, Nanjing 211171, P. R. China

Tel: +86-25-86178261

Fax: +86-25-86178261

E-mail: [duanhaibao4660@1](mailto:xmren@njtech.edu.cn)63.com

**1. Experimental**

**1.1. Chemicals and reagents**

All reagents and chemicals were purchased from commercial sources and used without further purification.

**1.2. Physical measurements**

Elemental analyses (C, H and N) were performed with an Elementar Vario EL III analytical instrument. IR spectra were recorded on a Bruker Vector 22 Fourier Transform Infrared Spectrometer (170SX) (KBr disc). Thermogravimetric (TG) experiment was performed with a TA2000/2960 thermogravimetric analyzer from 30 to 600°C at a warming rate of 10 K/min under a nitrogen atmosphere, and the polycrystalline samples were placed in an aluminum crucible. Powder X-ray diffraction (PXRD) data for the as-prepared was collected using Bruker D8 Advance powder diffractometer operating at 40 kV and 40 mA with Cu K radiation λ = 1.5418 Å at ambient temperature. Temperature and frequency dependent dielectric constant measurements were carried out employing Concept 80 system (Novocontrol, Germany) and the ac frequencies span from 1 Hz to 10^7^ Hz. Proton conductivity meas-urements at various relative humidities were performed using a conventional three-electrode method on a CHI 660D electrochemical workstation, in which the reference electrode was shortened with an auxiliary electrode, and powdered polycrystalline pellets with a diameter of 13 mm and thicknesses of roughly 1.35 mm. The frequency of the applied alternating current (ac) field ranged from 100 Hz to 2 MHz with 5 mV of signal amplitude. The DC offset was zero. Two copper plate electrodes were used to clamp the pellet sample, which was then suspended in saltwater solution in a glass bottle with a rubber plug. This bottle was put into an environmental test box. The RH was adjusted by the content of salt in the salt solution and measured using a moisture meter.

**X-ray crystallography.** Selected crystals of **1** at room temperature were centered on an Oxford Diffraction Xcalibur diffractometer equipped with a Sapphire 3 CCD detector and a graphite monochromated Mo Kα (λ = 0.71073 Å). The data collection routine, unit cell refinement, and data processing were carried out with the program CrysAlis. Structures were solved by the direct method and refined by the full-matrix least-squares procedure on F^2^ using SHELXL-97 program. The non-Hydrogen atoms were anisotropically refined using the full-matrix least-squares method on F^2^.

**1.3. Preparations for 1**

DABCO ( 0.224 g, 2 mmol ), CoCl_2_ ·6H_2_O ( 0.238 g, 1 mmol ), Me_2_HPO_3_ ( 0.5 mL ) and fused choline chloride / 1,3-dimethyl urea ( molar ratio 1 : 2 ) eutectic solution ( 2 ml ) were mixed, and the mixture was added to the reactor with glass lining. After ultrasonic shock for half an hour, the reactants were fully contacted and placed in the oven at 120 °C for 2 days. Purple crystals were obtained by washing with deionized ethanol aqueous solution ( volume ratio 1 : 1 ) before solidification and vacuum drying for two hours.


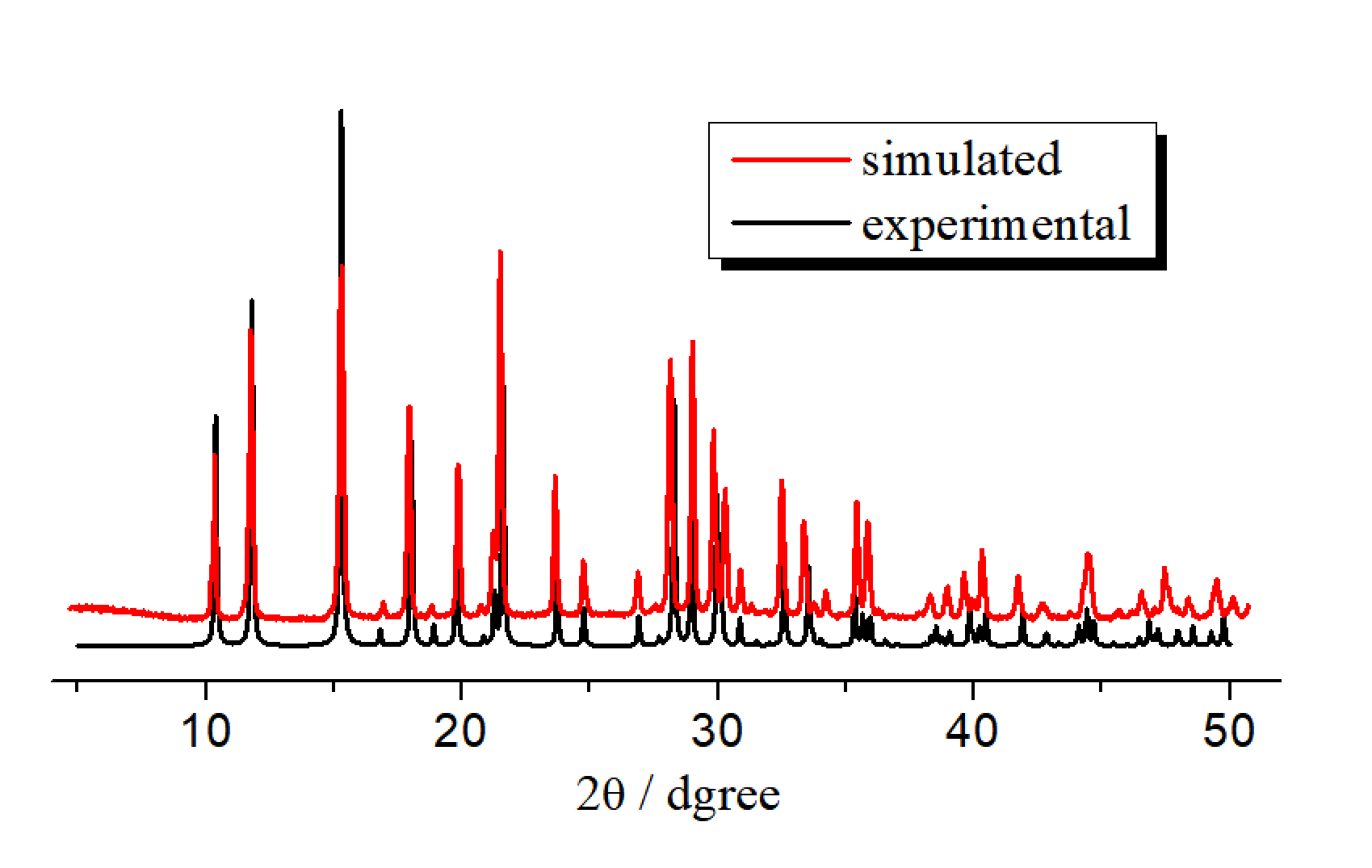


Figure S1 Powder X-ray diffraction patterns for as-prepared sample of **1** confirming the phase purity of the as-prepared sample (Black lines: experimental patterns; red lines: simulated profiles).


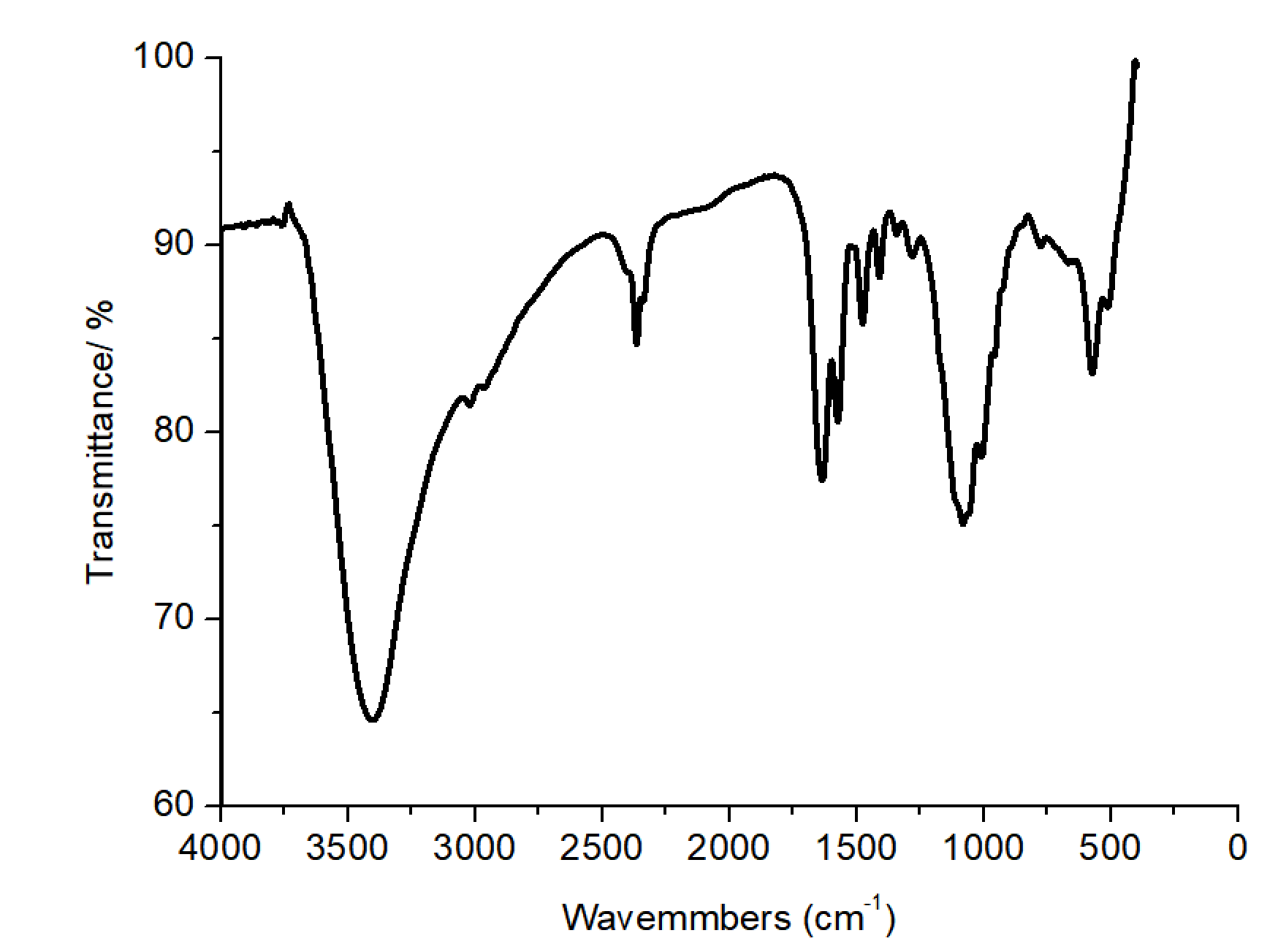


Figure S2 IR spectra of **1**


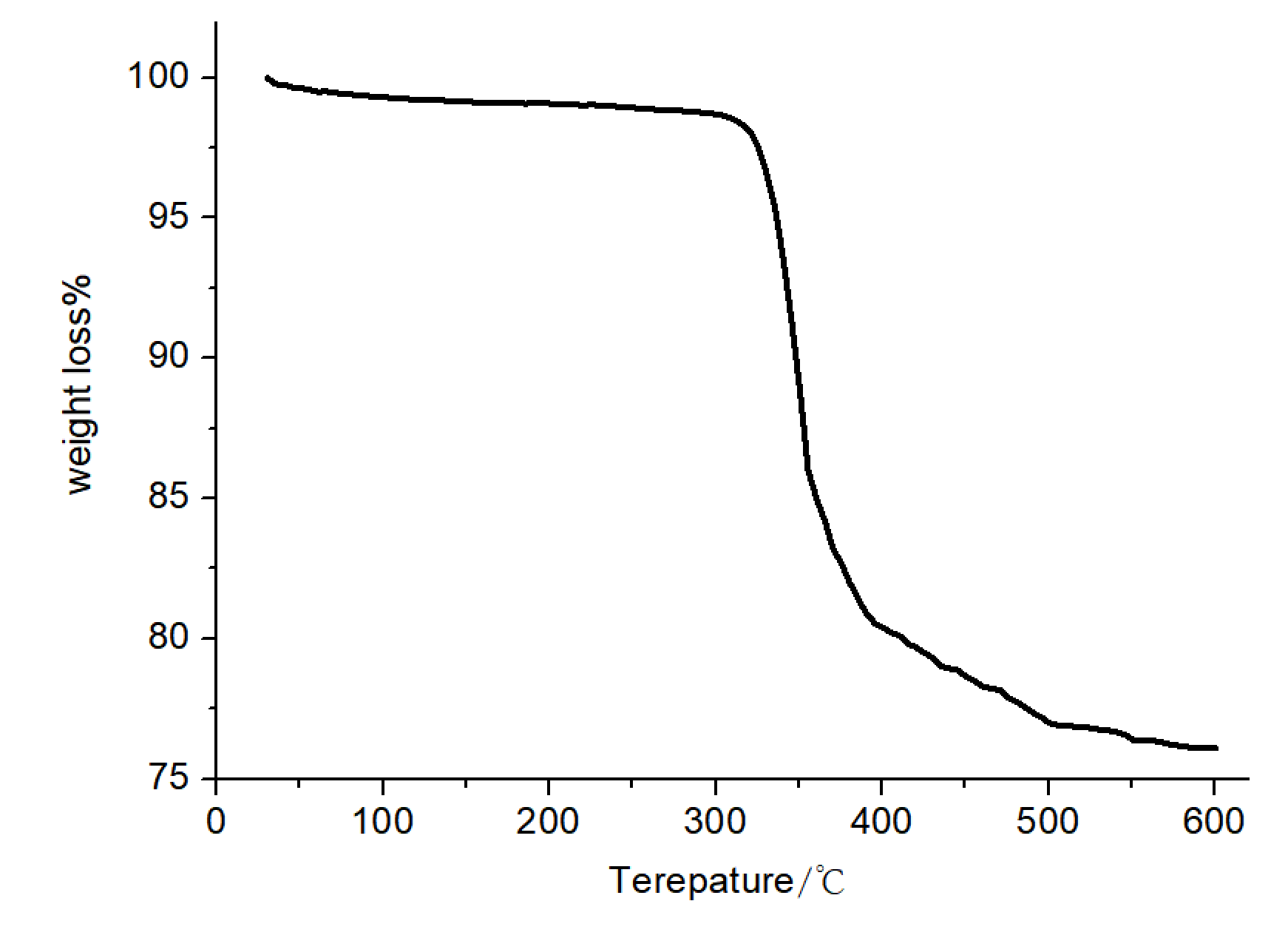


Figure S3 TG plot under N_2_ atmosphere for **1**


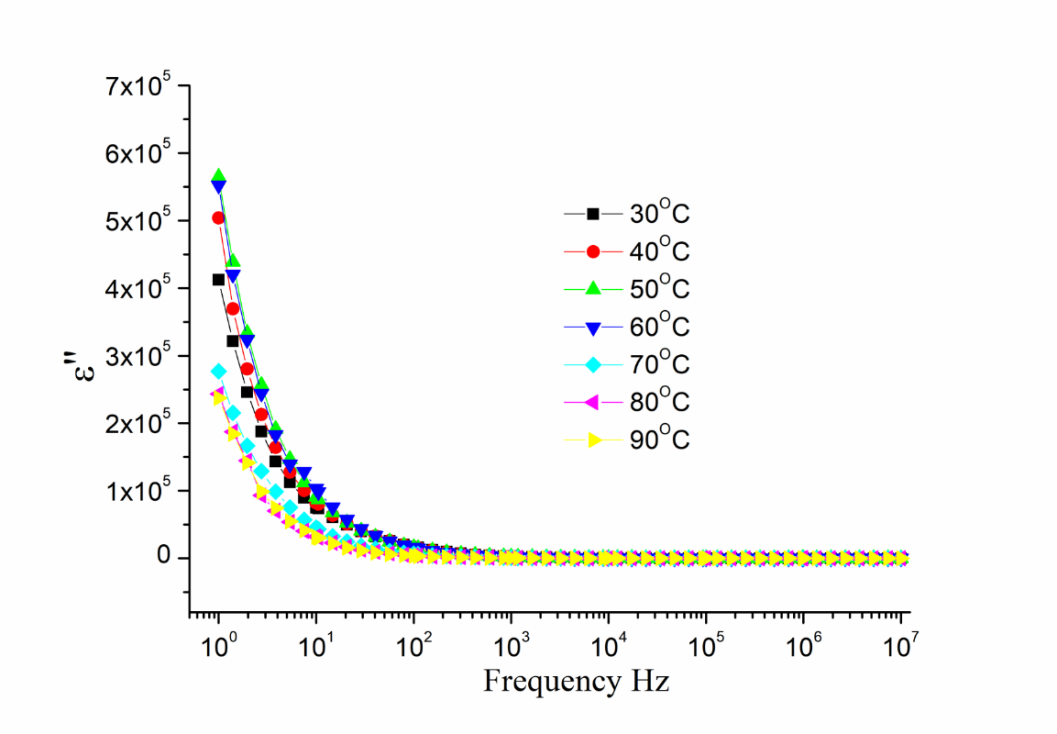


Figure S4 Frequency dependence of dielectric permittivity imaginary part (ε′′) of **1**


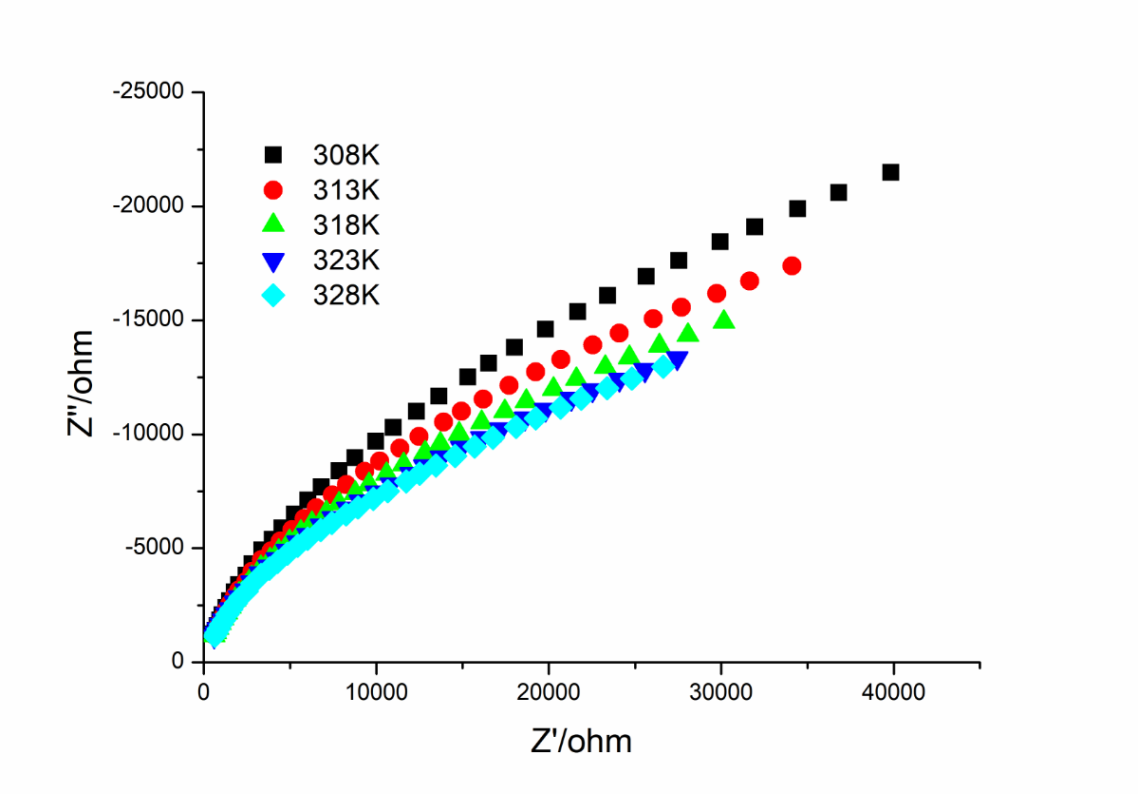


Figure S5 Nyquist plots of **1** at 60% RH at the selected temperatures
